# Impact of microscopic extrathyroidal extension on differentiated thyroid cancer post-surgical risk of recurrence: a retrospective analysis

**DOI:** 10.1007/s40618-023-02070-y

**Published:** 2023-03-28

**Authors:** L. Patti, S. Gay, L. Musso, C. Maltese, B. Spina, M. Minuto, S. Morbelli, L. Vera, M. Boschetti, D. Ferone, M. Albertelli

**Affiliations:** 1 Endocrinology Unit, Department of Internal Medicine and Medical Specialties (DIMI), University of Genova, Viale Benedetto XV, 6, 16132 Genoa, Italy; 2 Endocrinology Unit, IRCCS Ospedale Policlinico San Martino, Genoa, Italy; 3 Hospital Anatomic Pathology Unit, IRCCS Ospedale Policlinico San Martino, Genoa, Italy; 4 Endocrine Surgery Unit, IRCCS Ospedale Policlinico San Martino, Genoa, Italy; 5 Nuclear Medicine Unit, IRCCS Ospedale Policlinico San Martino, Genoa, Italy; 6 Department of Surgical Sciences (DISC), University of Genoa, Genoa, Italy; 7 Department of Health Sciences, University of Genoa, Genoa, Italy

**Keywords:** Differentiated thyroid cancer, Microscopic extrathyroidal extension, Recurrence risk, Predictive performance

## Abstract

**Purpose:**

In the last edition of the American Joint Committee on Cancer (AJCC) staging system, differentiated thyroid cancers (DTC) showing microscopic extrathyroidal extension (mETE) are considered comparable to intrathyroidal cancers for their clinical behavior and prognosis. The aim of the study is to evaluate the impact of this updated assessment of T, when applied to the postoperative recurrence risk stratification, according to the American Thyroid Association Guidelines (ATA-RR).

**Methods:**

One-hundred DTC patients who underwent total thyroidectomy were retrospectively evaluated. The downstaging of mETE was introduced in the definition of T, and the updated classification defined as modified ATA-RR (ATAm-RR). For each patient, post-surgical basal and stimulated thyroglobulin (Tg) levels, neck ultrasound (US) and post-ablative 131-I whole body scan (WBS) reports were considered. The predictive performance (PP) of disease recurrence was calculated both for each single parameter, as well as for all of them.

**Results:**

According to ATAm-RR classification, 19/100 patients (19%) were downstaged. ATA-RR proved a significant PP for disease recurrence (DR) (sensitivity 75.0%, specificity 63.0%, *p* = 0.023). However, ATAm-RR performed slightly better due to an increased specificity (sensitivity 75.0%, specificity 83.7%, *p* < 0.001). For both classifications, the PP was optimal when all the above-mentioned predictive parameters were considered.

**Conclusion:**

Our results suggest that the new assessment of T considering mETE resulted in a downgrading of ATA-RR class in a significant number of patients. This provides a better PP for disease recurrence, and the best PP was obtained when considering the whole predictive variables together.

**Supplementary Information:**

The online version contains supplementary material available at 10.1007/s40618-023-02070-y.

## Introduction

Differentiated thyroid cancers (DTCs) are the most common endocrine neoplasms, with a maximum incidence between 30 and 50 years of age and a higher prevalence in women, with a female-to-male ratio close to 3:1 [1, 2]. They are generally associated with a very low disease specific mortality and an excellent overall survival [3]. The diagnosis is mainly based on clinical examination, thyroid ultrasonography (US) and fine needle aspiration biopsy. Surgical approach is the first treatment, possibly followed by radioiodine (131-I) administration in selected cases [4].

Postoperative staging for thyroid cancer, as well as for other types of malignancies, is crucial to provide prognostic information, with the aim of planning an adequate surveillance and optimize the therapeutic strategy [4–6]. An accurate staging is based on risk stratification data, which can be obtained as part of preoperative testing, during surgery, or in the postoperative evaluation [4].

Over the years, multiple staging systems have been developed to predict the risk of mortality or recurrence in patients with DTC [7]. The TNM staging proposed by the American Joint Committee on Cancers (AJCC), in particular, is aimed at estimating the risk of disease related mortality [8]. In 2017, the AJCC staging system was updated to improve the survival predictive value. One of the main changes introduced by the new version focuses on the minimal extrathyroidal extension (mETE), which is now considered in the same way as an intrathyroidal disease, with respect to the assessment of T [9]. The mETE, indeed, is defined as a microscopically detected invasion of perithyroidal tissues, which seems not to have an impact on the clinical behaviour and prognosis of the disease [10].

Several studies confirmed the improvement brought by this last classification in terms of mortality risk stratification, compared to the previous one [8, 11, 12].

On the other hand, the American Thyroid Association (ATA) proposed a post-operative risk stratification system to predict recurrences, which includes the evaluation of T, together with molecular analysis (e.g., BRAF and TERT mutation) and histological features [i.e., the histological variant, the number and size of lymph-nodes involved]. Based on this, patients are stratified in three classes according to their risk of recurrence, providing indications about subsequent treatments [4, 13, 14].

The aim of the present study is to evaluate the impact of the updated assessment of T, as stated by the new AJCC classification as regards the mETE, when applied to the post-operative risk stratification system for disease recurrence (DR) proposed by the ATA.

## Materials and methods

### Study subjects and data collection

Among patients who underwent thyroidectomy between 2000 and 2015 and followed by the Thyroid Cancer multidisciplinary group of the IRCCS Policlinic University Hospital San Martino in Genoa (Italy), one hundred patients were randomly selected and retrospectively enrolled in this study. The inclusion criteria of the present study were the following: patients with histologically confirmed DTC [papillary thyroid carcinoma (PTC), follicular thyroid carcinoma (FTC) or their variants, Hurthle cell carcinoma], age ≥ 18 years, follow-up longer than 24 months and at least one evaluation per year for the whole follow-up period.

Medical and histological reports of all patients were reviewed, and the following data acquired: age at diagnosis, gender, histological subtype, extent of surgery (thyroidectomy or lobectomy), neck lymph node dissection (central and/or lateral), tumour size, number and region of lymph-nodes involved, post-surgical radioiodine ablation therapy (RAI), cumulative RAI dose, biochemical data [TSH, fT4, thyroglobulin antibodies (TgAb), post-surgical basal (psTg) and stimulated (stTg) thyroglobulin levels], imaging reports [US neck and whole body scan (WBS)].

### Study design

The post-surgical risk of recurrence was re-assessed for each patient, by reviewing all the histological reports, according to ATA 2015 Guidelines. Persistent disease and DR were considered as the final outcome. Subsequently, a modified stratification of the risk of recurrence was attributed to each patient, introducing the downstaging given by the mETE in the definition of T according to the new AJCC TNM edition. The updated classification was defined as ATA modified risk of recurrence (ATAm-RR). PsTg, stTg, neck US and post ablative 131-I whole body scans (rxWBS) reports at baseline were also considered.

After the initial assessment, each patient underwent morphological and biochemical re-evaluations, performed every 6 months during the follow-up: all these data were reviewed to detect the persistence or recurrence of the disease. The rise > 1 ng/ml of a previously undetectable basal Tg levels, stTg > 10 ng/ml, neck US reports suggestive of malignancy and tracer uptake at the rxWBS are the parameters that were used to define the persistence or recurrence of the disease. All the findings suspicious for structural recurrences were confirmed by CT and/or cytology.

The predictive performance for DR was calculated for each single parameter, as well as for the whole parameters together. Data collection and statistical analysis were performed in compliance with the Helsinki Declaration, 1964 and informed consent was obtained from all patients.

### Laboratory evaluations

Serum Tg was assayed through immuno-chemiluminescence (Roche Diagnostics, Mannheim, Germany). Functional sensitivity of the method was ≤ 0.5 ng/ml. TSH and fT4 were measured by mean of ultrasensitive immuno-chemiluminescence methods (Roche Diagnostics). Normality ranges were 0.3–4.2 mIU/l for TSH, 9.3–17.0 ng/l for fT4. TgAb were determined through commercial assays (DiaSorin, Saluggia, Italy).

### Imaging

US neck examinations were performed by means of a conventional high-resolution device with a Echocolor-Doppler module equipped with a ML 4–13 linear probe working at 7.5 MHz (MyLab Five, Esaote Biomedica, Genoa, Italy). The finding of hypoechogenic round lymph-nodes or the presence of measurable hypo- or iso-echogenic tissue in the thyroid bed were considered as characteristics of suspicion for disease persistence/recurrence. FNA was performed in every such cases, to confirm the presence of recurrent/persistent disease.

### Statistical methods

Parametric distribution of the data was assessed through Kolmogorov–Smirnov test. Data are reported in the text as “median, range” if non-parametric, as “mean ± standard deviation” when parametric.

Chi-squared test was used to evaluate the association among non-quantitative variables and recurrences, whereas logistic regression was used to correlate these last with quantitative parameters. Cut-off values for the best sensitivity and specificity were calculated by mean of ROC curves.

Afterward, the multiparametric score, comprehensive of the variables, which resulted related to the risk of recurrence, was calculated attributing one point for each variable that exceeded the cut-off value previously calculated, and ROC curves were used to assess the sensitivity and specificity of this score as well. The association between quantitative non-parametric data and the ATA-RR class was assessed by mean of Kruskal–Wallis test.

Statistical analyses were carried out by mean of MedCalc Portable Launcher software, version 2.2.0.0; the same program was used to create all figures and graphs.

## Results

Of the 100 patients enrolled, 79 were female. The characteristics of patients included in our analysis are summarized in Table 1. The mean age of the study population was 52.4 (± 14.1). Two patients underwent total lobectomy, 98 patients total thyroidectomy alone, a lymph-node dissection of the central compartment of the neck (level VI) was associated to total thyroidectomy in 21 cases, and a central and lateral neck compartment dissections (levels II–III–IV and Va) were performed in 10 patients. Final histology revealed a PTC in 93 patients: 84 were classic variant (25 microcarcinomas (mPTC)) and a 9 follicular variant (2 microcarcinomas). A follicular thyroid cancer was diagnosed in 5 patients. Two patients displayed both a PTC and an FTC.

**Table 1:** Table reporting the distribution of demographic, histopathological data and the risk of mortality and recurrence at the time of primary treatment in the study population

Characteristics	No. of patients/value
**Gender**	
Female	79
Male	21
**Age at diagnosis**	
Mean	52.4 (± 14.1)
**Histology**	
PTC (classic/follicular variant)	93 (84/9)
Follicular thyroid carcinoma	5
PTC + FTC	2
**Extent of surgery**	
Total thyroidectomy	98
Lobectomy	2
**Lymph node dissection**	
No	79
Central compartment neck dissection	11
Central + lateral compartment neck dissection	10
**Lymph nodes involvement**	
Present	19
Absent	81
**Neck region involvement**	
A3	5
A4	3
A6	13
**Stage**	
I	70
II	26
III	4
**ATA 2015 risk stratification system**	
Low risk	60
Intermediate risk	27
High risk	13
**ATA modified risk stratification system**	
Low risk	79
Intermediate risk	8
High risk	13

Median tumour size was 12 mm (1–45 mm). A lymph-nodes involvement was demonstrated in 19 patients, with a median amount of 2 (1–12) pathological lymph-nodes. In this regards the most affected neck region was the central compartment (A6) in 11/19, while lower lateral compartment (A4) and medium lateral compartment (A3) were involved in 2 and 4 cases, respectively. One patient reported pathological findings both in A4 and A6 dissection and another in A6 and A3.

Seventy-eight patients underwent 131-I radioiodine treatment after surgery, and the median dose administered was 80 mCi (range 30–120 mCi).

According to 2015 ATA Guidelines, post-surgical risk of recurrence (2015 ATA-RR) was low (LR) in 60, intermediate (IR) in 27 and high (HR) in 13 cases.

**Fig. 1:**
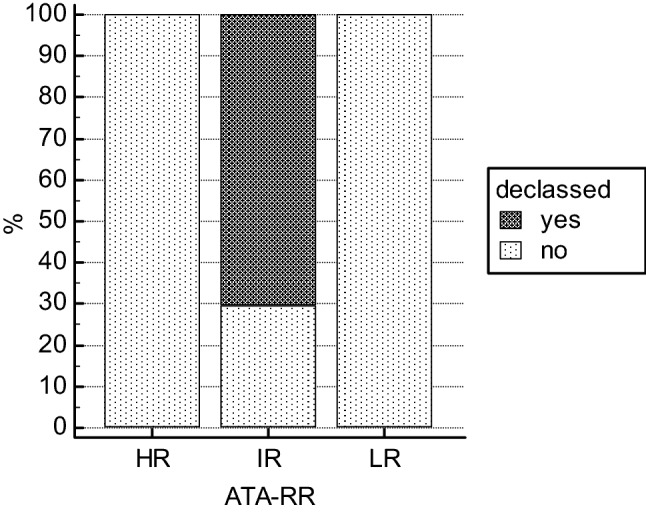
The graphic shows, for each ATA-RR class, the percentage of cases which changed their class after the updated assessment of T with respect to mETE

In the ATAm-RR, the proportions changed as follows: 79 patients resulted in LR class, 8 in IR and 13 in HR class. Therefore, 19% of patients were downgraded, all of them switching from the intermediate to the low-risk class (Fig. 1). In particular, 70.4% of the IR patients in our cohort were downstaged to the lower category.

Particularly, patients who were declassed displayed a change in the T assessment: 5 of them were reclassified as T1a, 11 as T1b, 1 T2a, and 2 as T2b.

Median psTg value was 0.48 ng/ml (0.04–48.00 ng/ml), while stTg was 2.90 ng/ml (0.04–140.00 ng/ml). Ninety-seven patients underwent post-surgical neck US examination. No patient showed gross persistent disease at the US examination. The post-dose rxWBS showed an uptake outside the thyroid bed in six patients.

Both 2015 ATA-RR and ATAm-RR class were significantly associated with psTg (*p* = 0.029 and *p* = 0.032, respectively) but not with stTg (*p* = 0.110 and *p* = 0.104).

The 2015 ATA-RR and ATAm-RR did not show any association with post-surgical US findings (*p* = 0.960 and *p* = 0.967).

A correlation between 2015 ATA-RR classification and rxWBS findings was demonstrated (*p* = 0.003), as well as between rxWBS and ATAm-RR (*p* = 0.002).

The average duration of the follow-up was 127.6 (± 52.7) months. During that period, a morphological thyroid cancer recurrence occurred in eight patients.

No statistically significant relationship was found between recurrence and age (*p* = 0.651), gender (*p* = 0.871), the presence of detectable TgAb levels (*p* = 0.817), histology (*p* = 0.505), or the type of surgery performed (*p* = 0.059).

Tumour size showed a significant correlation with disease recurrence (sensitivity 75.0%, specificity 68.1%, *p* = 0.014, cut-off > 17 mm). The presence of pathological lymph-nodes was correlated with recurrences (*p* = 0.044): specifically, they proved more frequent when A6 + A3 (1/1), A4 (1/2) and A6 (2/11) were involved (*p* < 0.001). The number of pathological lymph-nodes was associated with disease recurrence, as well (sensitivity 57.1%, specificity 90,7%, *p* = 0.047, cut-off > 1).

The 2015 ATA-RR showed a significant predictive performance for recurrences (sensitivity 75.0%, specificity 63.0%, *p* = 0.023), but ATAm-RR performed slightly better due to an increased specificity (sensitivity 75.0%, specificity 83.7%, *p* < 0.001).

Post-surgical neck US findings (*p* = 0.873) and the rxWBS (*p* = 0.871) were not predictive of recurrence. On the other hand, stTg seemed to perform better than basal psTg, at least in this setting (*p* = 0.004 vs *p* = 0.043).

**Fig. 2:**
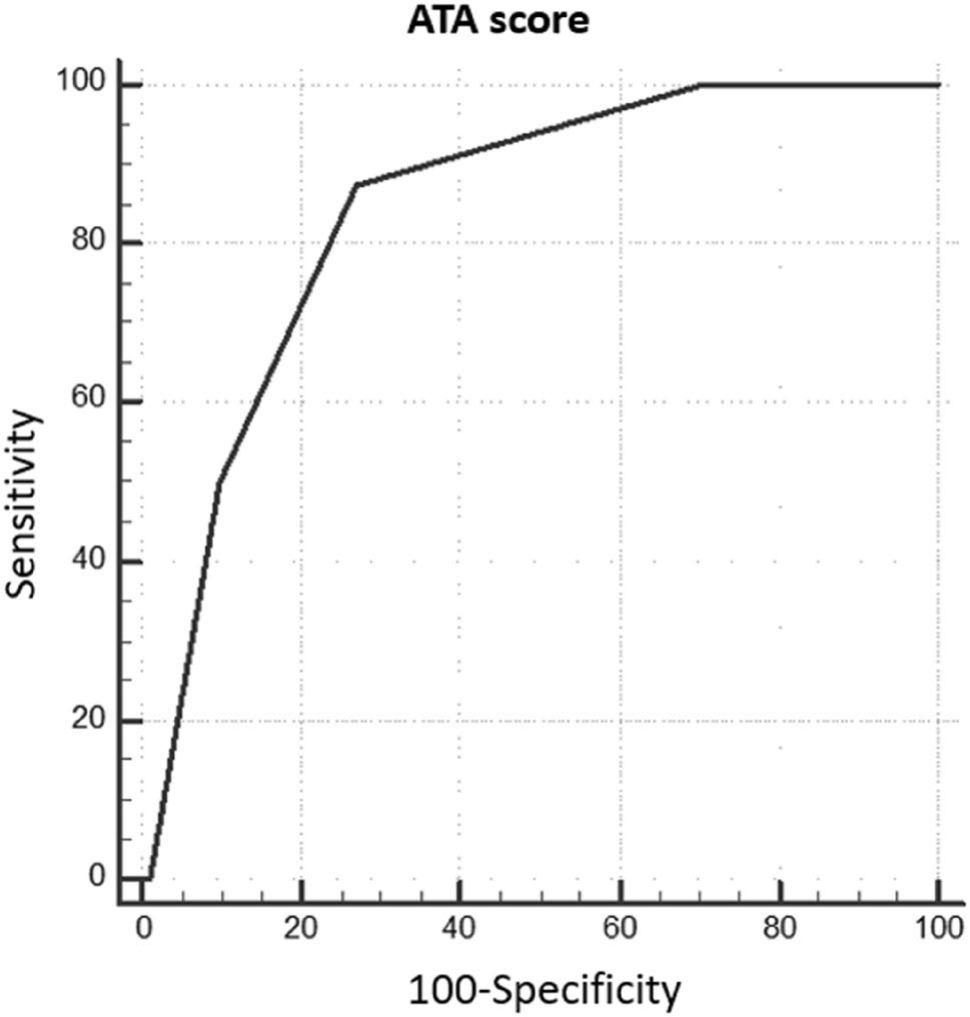
ROC curves representing the predictive performance for DR of the cumulative score calculated through all the parameters considered (pathological or suspicious US findings, basal psTg ≥ 1 ng/ml, stTg ≥ 10 ng/ml) together with 2015 ATA-RR

**Fig. 3:**
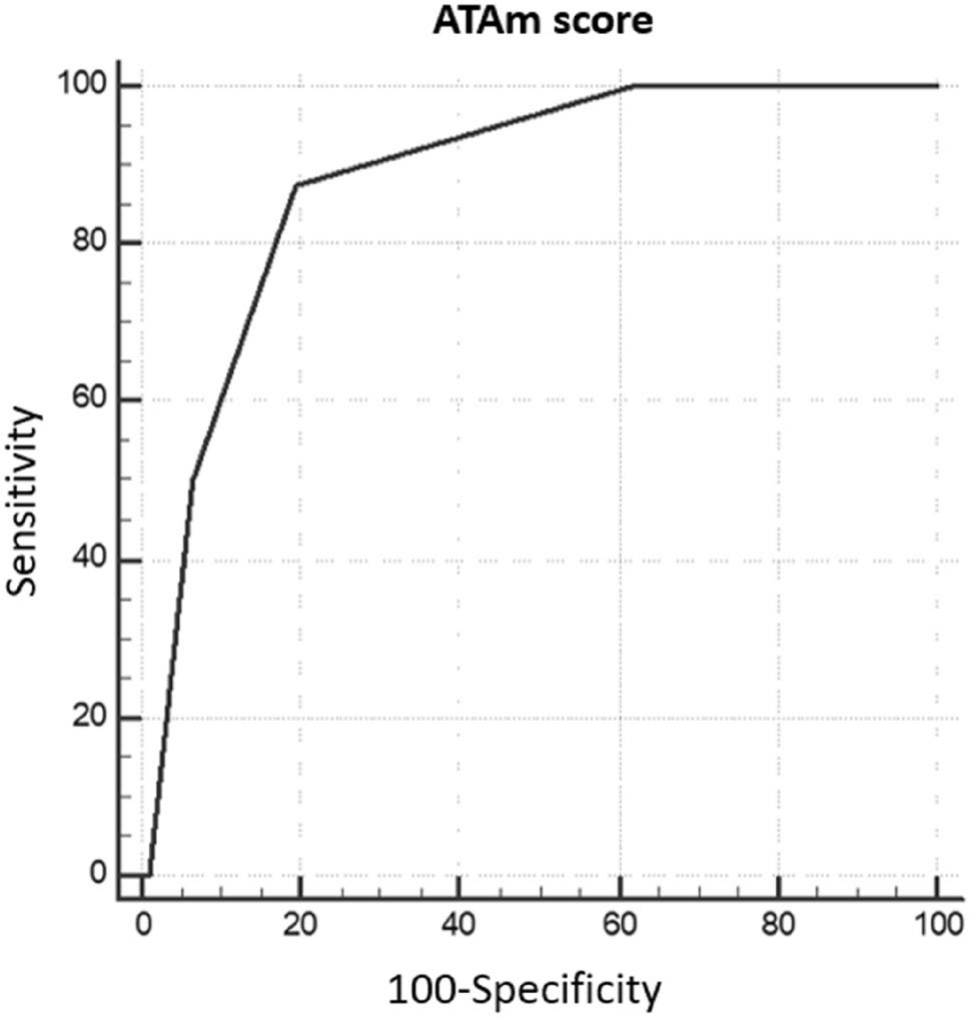
ROC curves representing the predictive performance for DR of the cumulative score calculated through all the parameters considered (pathological or suspicious US findings, basal psTg ≥ 1 ng/ml, stTg ≥ 10 ng/ml) together with 2015 ATAm-RR

Nevertheless, the best predictive performance was obtained when all the above-mentioned variables were considered (2015 ATA-RR, pathological or suspicious US findings, basal psTg ≥ 1 ng/ml, stTg ≥ 10 ng/ml): in this scenario, the adoption of ATAm-RR slightly improved the specificity of the assessment (ATAm-RR sensitivity 87.5%, specificity 80.4%, *p* < 0.001 versus sensitivity 87.5%, specificity 72.8%, *p* < 0.001 considering 2015 ATA-RR) (Figs. 2, 3).

When the evaluation of tumour diameter (> 17 mm) and the number of involved lymph-nodes (> 1) were considered in addition to all the other variables into this comprehensive assessment, they improved its specificity while reducing its sensitivity, and differences in specificity between ATA-RR and ATAm-RR were maintained (sensitivity 75.0%, specificity 84.8%, *p* < 0.001 considering 2015 ATA-RR and sensitivity 75.0%, specificity 88.0%, *p* < 0.001 with ATAm-RR).

## Discussion

The present study was designed to evaluate the impact of the new assessment of T, with respect to mETE, on post-operative ATA 2015 risk stratification. To the best of our knowledge, very few studies specifically addressed this issue to date, and our results were able to provide new evidence about a better predictive performance for recurrences.

Despite the low numerosity of the sample considered, we were able to find some significant associations which could provide interesting insights both for clinical practice in predicting the risk of recurrence and could inspire further research on this topic.

Historically, ETE has been recognized as a potential negative prognostic factor in DTCs [15], and it has often been included in thyroid cancer staging systems, i.e. MACIS (metastasis, age, completeness of resection, invasion, and size) [16], AMES (age, distant metastasis, extent and size of primary cancer) and GAMES (tumour grade, age, metastases, extent, and size) [17].

On the other side, the impact of a mETE has been questioned, and recent investigations have showed its negligible effect on relapse-free survival rates [18, 19]. Thus, the 8th last edition of the AJCC staging system for DTC has removed it from the definition of pT3 disease [20].

In line with this evidence, our results suggest that the downgrading of T in DTCs displaying mETE, applied to the post-operative ATA risk of recurrence, provides a better predictive performance for recurrences, due to an increased specificity (*p* < 0.001). Therefore, tumours with mETE might be assimilated, in term of prognosis, to intrathyroidal cancers.

According to this concept, several studies [21–23] have previously showed that mETE is not associated with higher rate of recurrence compared with those without ETE. In particular, Ito et al. and Woo et al. have reported that the presence or absence of mETE did not significantly influence recurrence-free survival among patients with solitary PTC or microcarcinoma. On the other hand, two studies from Tran et al. and Park et al. suggest that mETE is a parameter that might negatively affect the prognosis of thyroid cancer [24, 25], but no multivariate analysis was carried out to support this evidence [20].

As a matter of fact, in the clinical practice, the initial estimation of the risk of recurrence is routinely and dynamically re-assessed during the follow-up, as well as treatment response, mainly through psTg and stTg levels, neck US and post ablative 131-I WBS [4, 26]. Regarding Tg, several studies appear to corroborate our findings and emphasised that a low psTg level could be considered as a favourable prognostic factor for patients with DTC [27–29]. In line with these results, a meta-analysis conducted by Giovanella et al. in 2014 confirmed psTg testing as a readily available and reliable tool, with a high negative predictive value (NPV) toward cancer recurrences [30]. Furthermore, psTg level might be an independent postoperative assessment factor and provide ongoing serologic evidence for the recurrence risk stratification system [31].

Conversely, in our population, neither post-surgical rxWBS nor neck US, when taken alone, showed a correlation with the risk of recurrence, and this is in accordance with Klain et al. who found that rxWBS findings did not bring any significant incremental value in the identification of patients at higher risk of recurrence in low-risk cohort of patients [32]. Although US reports are reliable and relevant methods for the early diagnosis and follow-up, they seem not to provide sufficient information to ascertain the risk of thyroid cancer recurrence in the neck region [33].

In this study, we found no association between recurrences and the age or gender of the patients (*p* = 0.651 and *p* = 0.871, respectively), and this was consistent with other studies in literature [34]. Nevertheless, it is well recognized that an older age is associated with a poorer prognosis: for this reason, the 8th edition of the TNM system, raised the cut-off for an increased risk of death from 45 to 55 years [35]. This update was supported by a large retrospective study from 10 American institutions [36].

On the other hand, gender is not considered as a prognostic factor in the commonly used staging systems, and this study, as well as some other reports, showed that the overall survival is not affected by the gender at birth [37, 38]. Nevertheless, this topic is still matter of debate, as other studies reported a higher risk of DTC recurrence in men than in women [39, 40].

The retrospective design and the relatively small sample size represent a limitation of the present study. However, the availability of a long and accurate follow-up, with multiple annual evaluations performed and registered within the same centre, partially counterbalance the relatively small cohort evaluated, reducing the intra-observer biases and improving data reliability.

## Conclusions

The present findings suggest that the adoption of ATAm-RR, compared to 2015 ATA-RR, is able to improve the predictive performance of DR due to an increased specificity. Furthermore, the majority of patients classified as IR in our study population was declassed using ATAm-RR. The best predictive performance was obtained when considering the whole abovementioned variables (2015 ATAm-RR, pathological or suspicious US findings, basal Tg ≥ 1 ng/ml, stTg ≥ 10 ng/ml) together.

The update introduced by the 8th edition of AJCC confirms to be a significant step forward in initial risk stratification for patients with DTC and represents an important tool to improve the personalized treatment of thyroid cancer.

Despite the low numerosity of the population which has been investigated, our research was able to provide statistically significant data: in this perspective, subsequent prospective studies performed on extended cohorts of patients might be advisable to provide more solid evidences on this intriguing topic.

### Supplementary Information

Below is the link to the electronic supplementary material.

Supplementary file1 (XLSX 30 KB)
